# Validated DNA isolation method ensuring successful long-read sequencing of cattle semen genome

**DOI:** 10.1371/journal.pone.0308011

**Published:** 2024-08-07

**Authors:** Erwan Denis, Cécile Grohs, Cécile Donnadieu, Carole Iampietro

**Affiliations:** 1 INRAE, Castanet-Tolosan, France; 2 INRAE, AgroParisTech, GABI, Université Paris-Saclay, Jouy-en-Josas, France; Universite Clermont Auvergne, FRANCE

## Abstract

Obtaining high-quality DNA suitable for long-read sequencing can be difficult for many types of tissues and cells, and it is a key step in current genomic studies. The challenge is even greater when it comes to isolating genomic DNA from mammalian spermatozoa, as DNA is tightly packed into a cell with a robust membrane rich in disulfide bonds. Here we describe a method for isolating high molecular weight DNA from Bovine commercial semen straws. This protocol includes a cleaning step to remove diluents and preservatives used for the long-term storage of the semen, which may affect long read sequencing. It is based on a simple salting-out method and avoid the use of spin columns, strong mixing or intensive centrifugation, in order to limit DNA fragmentation. However, we have adapted this protocol to facilitate the disruption of cell membranes and disulfide bonds with strong chaotropic and reducing agents. The average size of the fragments produced was approximately 49 kb, ranging from 25 to 85 kb, according to the femto pulse profiles.This method was used to isolate DNA from semen straws, more than 80 of them were successfully sequenced using the Continuous Long-Read (CLR) sequencing mode on the PacBio SequelII platform to study genome diversity and notably to detect large structural variations within genomes.

## Introduction

Today, long fragment sequencing has become the technology of choice to study genomes. Long reads facilitate genome assembly and make them more contiguous, often reaching a chromosomal level. It also facilitates the detection of large structural variations. In particular, insertions are difficult to highlight using short reads, whereas reading a long native DNA molecule makes it easy to identify precise insertion points within the genome. To clarify the role and importance of this polymorphism within the bovine population, we have sequenced DNA isolated from frozen semen on the long read sequencing platform PacBio SequelII. Commercial semen straws represent an important source of bovine genetic material, easily available because they are widely used for animal insemination and can be kept for many years after the animal has been culled.

Therefore, we developed a protocol (based on those previously published [1–3]) that enables us to perform a large number of DNA isolations from commercial semen straws, quickly (4h30) and easily, since all the solutions are commercially available.

A semen straw is a breeding stock containing sperm from a single male, diluted in a nutrient and preservative solution that allows long-term storage in liquid nitrogen. There is no universal standard for semen straw production, so the amount of sperm present in the straw varies. As a result, it is sometimes necessary to use more than one straw to obtain sufficient DNA for downstream sequencing.

In addition, the conditioning and storage of the straw has been optimized over time to ensure fertilization. Because each company makes its own choices, the composition of the diluent used for preservation is often unknown and may vary from straw to straw, depending on the supplier or the date of collection. Given this situation, we first wash the frozen semen with 1X PBS to remove potential contaminants that could interfere with sequencing, especially by causing low data throughput (as suggested in Hossain *et al*.). For applications that require complete removal of somatic cells present in the semen, this washing step will not be sufficient. In this case, other strategies, like gradient centrifugation [3, 4], can be used to recover only sperm cells. Since our goal is to obtain large fragments, it is important to handle the DNA gently and not use methods that could fragment it, such as mechanical shearing. We have therefore chosen a salting-out method that combines the advantages of not using organic solvents, purification columns or excessive agitation (as described further).

The second step of the protocol involves unpacking DNA from the sperm cells and cells lysis. Sperm DNA is compactly retained in nucleus thanks to the presence of protamines linked together by disulfide bonds. Dithiothreitol (DTT) and 2-mercaptoethanol are both widely used in DNA extraction methods as powerful reducing agents that cleave disulphide bonds. However, tris(2-carboxyethyl)phosphine (TCEP) has been shown to be more effective than DTT in reducing disulphide bonds [5]. In addition, TCEP is odourless, more stable than DTT and resistant to oxidation [5]. Therefore, after the washing step, we preferred to use TCEP, combined with the action of the guanidine thiocyanate, a strong chaotropic agent (included in RLT Buffer from Qiagen), to start cell lysis, as in Wu *et al*.

After the initial action of RLT-TCEP mixture, we add the commercial Cell Lysis Solution to the mix (from Qiagen Puregene Tissue Kit). We have observed that lysis is enhanced by the first RLT-TCEP step as well as by the addition of Proteinase K (PK) to the Cell Lysis Solution. PK digests nuclear proteins and homogenises the extract. Unlike Wu *et al*., we did not use beads and agitation for homogenization in order to preserve DNA integrity.

In our protein removal process, we use the Protein Precipitation Buffer from the Qiagen Puregene Tissue Kit, avoiding the phenol-chloroform method [4, 6] due to concerns about the use of organic solvents known to be toxic. In addition, this choice was made to minimize the need for vigorous mixing and centrifugation, and to protect against potential DNA fragmentation as reported in the study by Hossain *et al*.

Instead of column-based DNA isolation [3], our approach involves DNA precipitation using isopropanol. While column methods are known to yield high quality DNA, they fall short in recovering a sufficient amount of high molecular weight molecules critical for long-read sequencing, especially when working with limited amounts of material. Our choice of isopropanol precipitation overcomes this limitation and ensures improved recovery of the high molecular weight DNA required for long-read sequencing. However, DNA can be difficult to dissolve after isopropanol precipitation and requires careful handling. The key to proper dissolution and preservation of DNA in the final step of washing the DNA pellet with ethanol is to not centrifuge at too high a speed (5000g maximum) and to never dry the pellet completely. Despite these precautions, DNA can still be difficult to resuspend. In this case, DNA in EB buffer can be heated to 60°C for one hour to dissolve it or placed on a rotating wheel at 4°C for several hours (overnight or more). It is recommended not to freeze the DNA to preserve long fragments and to store it at 4°C until further processing.

## Materials and methods

The protocol described in this peer-reviewed article is published on protocols.io https://dx.doi.org/10.17504/protocols.io.j8nlkw1qwl5r/v1 and is included for printing as supporting information file 1 with this article.

### Expected results

**Fig 1:**
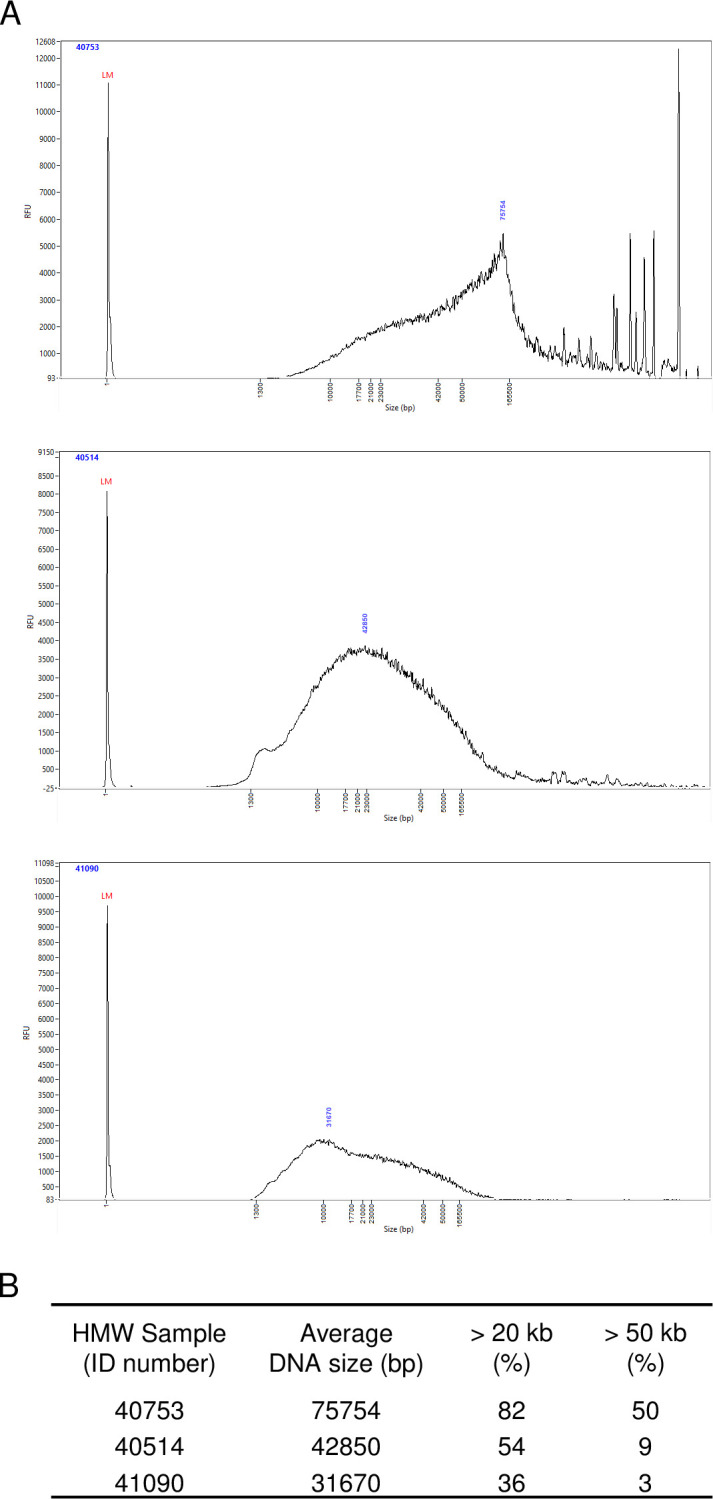
DNA fragment length distribution after DNA isolation. (A) DNA fragment length distribution for 3 representative samples (Fragment size distributions were assessed using the Femto pulse Genomic DNA 165 kb Kit (Agilent). LM = Lower Marker. (B) Average DNA fragment sizes and optical density ratios (260/280 and 260/230) for the 3 samples above.

Using this protocol, we expect to obtain large DNA fragments suitable for long read sequencing. To check the distribution of the different fragment lengths obtained, 37 of the 84 DNAs were randomly selected and analyzed using femto pulse (Agilent). The average size of the fragments generated is around 49 kb, ranging from 25 to 85 kb. As already mentioned above, the commercial semen straws that we used can vary greatly from one to another (different source of supply, storage condition and time preservation). We believe that these differences between straws lead to the various profiles we obtained. We illustrated the variability in DNA fragment size distribution in Fig 1, by showing 3 representative samples. All the 37 profiles are shown in S1 Fig.

When the proportion of small DNA molecules is very high, as in sample 3 (Fig 1), we used the BluePippin size selection system (SAGE Science) to eliminate these small molecules, as recommended by PacBio.

Since storage and preservation conditions, as well as the quantity of material present in each straw, may vary, the amount of DNA obtained varies. Table 1 shows the minimum, maximum, and average DNA quantities obtained from 84 semen samples.

**Table 1:** Summary of DNA isolation results and run outputs.

	Quantities (μg)	Concentration (Qubit) (ng/ μl)	Volume (μl)	OD Ratio 260/280	OD Ratio 260/230	Number of Gbases
**Mean**	23	141	273	1.84	0.52	143
**Max**	79.5	792	750	2.17	2.13	228
**Min**	4.3	13	50	1.13	0.10	80

Quantities, concentrations, volumes, OD ratios and single CLR run outputs obtained after HPM extraction vary from one sample to another within the same experiment. The Mean line indicates the mean values obtained for 84 samples. The Max and the Min lines indicate the highest and the lowest values obtained respectively for each measurement. All sample values are available in S1 Table.

S1 Table shows the amount of DNA obtained for all the 84 samples along with nanodrop absorbance ratios and sequencing output.

It is important to note that the absorbance ratio may not always meet expectations (i.e the 260/280 ratio ~1.8 and the 260/230 ratio ~2), especially for the 260/230 ratio. This discrepancy is most likely due to the presence of compounds from Cell Lysis Solution which absorb in the UV range. In our experience, this low ratio had no negative impact on the quantity or quality of the data obtained by sequencing on PacBio SequelII. Moreover, one DNA semen sample have been sequenced on Nanopore GridION platform for a few hours to see if we could observe the pore-blocking effect often seen with poor quality DNA. This short sequencing showed that sequencing processed on GridION went fine (data not shown), and this make us believe that our isolation method can be used for Nanopore sequencing.

Analysis of PacBio CLR sequencing results has shown that our protocol can provide homogeneous coverage across the bovine genome (data not shown) and can detect structural variants. Indeed, of all the long-read sequencing data generated using our protocol, some have been used to validate putative interchromosomal rearrangements detected on the basis of genotyping data analysis [7]. These sequencing data have already been published and are accessible on the European Nucleotide Archive (ENA, Project: PRJEB59364) [7]. All the additional sequencing data from more than one hundred bovine semen DNA isolated with our method will be submitted to ENA and included in the umbrella project PRJEB60075.

## Supporting information

S1 File Protocol from protocols.io in.pdf format. (PDF)

S1 Fig Overlay of all 37 Femto pulse DNA profiles. DNA fragment length distribution for 37 randomly selected samples (Fragment size distributions were assessed using the Femto pulse Genomic DNA 165 kb Kit (Agilent). Four different panels showed overlapping profiles of various DNA samples.
(TIF)

S1 Table DNA isolation results and run outputs of the 84 samples. Average size, volume, concentrations, OD ratios, quantities, and sequencing output (Gbases/SMRT cell) obtained for 84 samples. ND: No data.
(TIF)
