# Supplementary material for: Validated DNA isolation method ensuring successful long-read sequencing of cattle semen genome
Source: PLoS One. 2024 Aug 7;19(8):e0308011. doi: 10.1371/journal.pone.0308011 (PMC11305549; doi:10.1371/journal.pone.0308011)
Supplement: S1 File — (PDF) [file pone.0308011.s001.pdf]

Jan 18, 2024

DNA isolation from cattle semen for long read sequencing

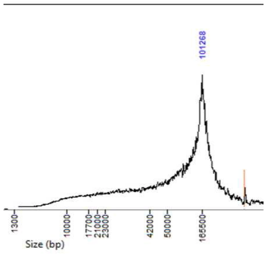

DOI
dx.doi.org/10.17504/protocols.io.j8nlkw1qwl5r/v1

Erwan Denis[1], Cecile Grohs[2], Carole Iampietro[1]

[1]INRAE, US 1426, GeT-PlaGe, Genotoul, France Genomique, Université Fédérale de Toulouse, Castanet-Tolosan, France;
[2]Université Paris-Saclay, INRAE, AgroParisTech, GABI, 78350, Jouy-en-Josas, France

Carole Iampietro
INRAE, GeT-PlaGe, Genotoul, France

OPEN ACCESS

DOI: dx.doi.org/10.17504/protocols.io.j8nlkw1qwl5r/v1

Protocol Citation: Erwan Denis, Cecile Grohs, Carole Iampietro 2024. DNA isolation from cattle semen for long read sequencing. **protocols.io https://dx.doi.org/10.17504/protocols.io.j8nlkw1qwl5r/v1**

License: This is an open access protocol distributed under the terms of the **Creative Commons Attribution License**, which permits unrestricted use, distribution, and reproduction in any medium, provided the original author and source are credited

Protocol status: Working
We use this protocol and it's working

Created: March 24, 2023

Last Modified: January 18, 2024

Protocol Integer ID: 79397

Keywords: extraction, high molecular weight, sperm, DNA, Long read sequencing, bovine, DNA isolation, PacBio

Funders Acknowledgement:
European Union and Occitanie region
Grant ID: Operational Program FEDER-FSE MIDI-PYRENEES ET GARONNE 2014-2020

Abstract

Here we describe a method for isolate high molecular weight DNA from commercially available frozen bull semen straws.
This protocol is based on a salting-out method and uses several commercially available solutions. It consists of several steps: washing of semen, lysis, removal of proteins and precipitation of genomic DNA.
This protocol was used to isolate DNA from sixty semen straws, all of which were successfully sequenced using the CLR sequencing mode on the PacBio SequelI platform.

Guidelines

Salting out is a good method to obtain high molecular weight (HMW) DNA, as it avoids damaging steps such as the use of purification columns or heavy mixing with phenol/chloroform.
Note that all mixing steps should be gentle to obtain HMW DNA fragments (from lysis steps to DNA precipitation). We also recommend to use DNA low bind tubes.

Protocol materials

- Proteinase K **Qiagen Catalog #**19133 Step 5
- Tris(2-carboxyethyl)phosphine hydrochloride solution **Merck MilliporeSigma (Sigma-Aldrich) Catalog #**646547-10X1ML Step 1
- Buffer RLT **Qiagen Catalog #**79216 Step 1
- Phosphate-buffered saline, pH 7.4 Step 2
- DNA LoBind Tubes 2.0 mL **Eppendorf Catalog #**30108078 Step 2
- EB buffer **Qiagen Catalog #**19086 Step 9
- Isopropanol Step 8
- Puregene Tissue Kit **Qiagen Catalog #**158063 Step 5

Safety warnings

See Safety Data Sheets for warnings and safety hazards.

Before start

As we use commercial sperm straws to perform our extractions, we do not always know the composition of these straws, the quantity of material contained, the nature of the diluents and preservatives used. This is why it is sometimes necessary to use several straws to obtain enough material for sequencing. It is also sometimes wise to perform several washes (see step 3) to eliminate contaminants from diluents and preservatives.

Preparation of reagents

1 Immidiately before use, prepare a mix containing RLT buffer (Qiagen) and TCEP [Tris(2-carboxyethyl)phosphine hydrochloride] to a final volume of 500µL per sample as follow:

- 450 µL RTL
- 50 µL TCEP

Buffer RLT **Qiagen Catalog #**79216

Tris(2-carboxyethyl)phosphine hydrochloride solution **Merck MilliporeSigma (Sigma-Aldrich) Catalog #**646547-10X1ML

Note

This mixture of a guanidine-based reagent (RLT) and a thiol-free reducing agent facilitate dissociation of disulfide bonds (Wu *et al*, 2018).
TCEP is odorless, and more stable than DTT (Han & Han, 1994).

CITATION

Han JC & Han GY (1994). A Procedure for Quantitative Determination of Tris(2-Carboxyethyl)phosphine, an Odorless Reducing Agent More Stable and Effective Than Dithiothreitol. Analytical Biochemistry.

LINK

https://doi.org/10.1006/abio.1994.1290

CITATION

Wu H, de Gannes MK, Luchetti G, Pilsner JR (2015). Rapid method for the isolation of mammalian sperm DNA..

LINK

https://doi.org/10.2144/000114280

Preparation of sample

2 **Recovery of spermatozoa from the straw:**

- Empty the 200 µL Sample in a 2 mL tube by cutting the two ends of the straw

DNA LoBind Tubes 2.0 mL **Eppendorf Catalog #**30108078

- Rince the straw it with 200 µL 1X PBS Room temperature

Phosphate-buffered saline, pH 7.4 **Contributed by users**

3 **Wash:** 10m

- Add 800 µL more PBS (up to 1 mL 1X PBS)
- Pellet 1000 x g, Room temperature, 00:05:00
- Discard the supernatant

Second wash is optional (no significant impact observed)

-Re-suspend in 1 mL 1X PBS

-Pellet 1000 x g, Room temperature, 00:05:00

-Discard the supernatant

Note

Centrifuge gently so that the pellet does not stick. It should be easy to resuspend for efficient lysis.

Lysis

4 **Step one:** 10m 10s

Add 500 µL of RLT-TCEP to the pellet

- Vortex 00:00:10 by pulsing at max speed
- If necessary, use a wide opening tip to resuspend the pellet
- Incubate On ice 00:10:00

5 **Step two:** continue with Qiagen Puregene Tissue kit adapted as follow 1h 30m

- Add 500 µL of Cell Lysis Solution
- Add 60 µL of [M] 20 mg/mL proteinase K (20 mg/ml)
- Mix by inversion (about 25 inversions)
- Incubate 55 °C 01:30:00

Puregene Tissue Kit **Qiagen Catalog #**158063

Proteinase K **Qiagen Catalog #**19133

6 **Remove RNA**: 16m

- 3 µL RNAse from Qiagen Puregene Tissue Kit

- Incubate 37 °C 00:15:00
- Incubate On ice 00:01:00

Protein precipitation

1m 15s

7 - Add 200 µL of Protein precipitation buffer (from Qiagen Puregene Tissue Kit) 6m 15s

- Mix by hand or gently vortexing 00:00:15
- Incubate On ice 00:05:00
- Centrifuge 16000 x g, Room temperature, 00:01:00

DNA precipitation

6m

8 - Transfert the supernatant to a new tube containing 600 µL of Isopropanol 6m

- Carrefully invert the tube 25-50X times to form the pellet
- Incubate 00:05:00 Room temperature
- Centrifuge 16000 x g, 00:01:00
- Discard supernatant

Isopropanol **Contributed by users**

9 - Add 600 µL of 70% ethanol to the pellet

- Centrifuge 5000 x g, 00:02:00
- Discard supernatant
- Almost dry the pellet Room temperature 00:05:00
- Add 50 µL to 100 µL of EB (Qiagen) or TE buffer to eluate DNA
- Store DNA at 4°C

EB buffer **Qiagen Catalog #**19086

Note

DNA in EB buffer can be heated to 60°C for 1 hour to dissolve it. Do not vortex or pipet DNA. It is recommended not to freeze the DNA to preserve long fragments.

Expected result

Of the 60 extractions carried out using this protocol, the average size of the fragments generated is around 53 kb, ranging from 25 to 120 kb on average. We expect 30 ug of DNA from a commercial semen straw, but this figure can vary considerably from sample to sample. We obtained absorbance ratios of 260/280 for DNA of around 1.8 nm, and 260/230 ratios averaging 0.5 nm. Low ratios have already been observed using RLT buffer (Wu *et al*, 2018), but these did not affect PacBio sequencing significantly.
Some of these DNA have been sequenced and published in Jourdain *et al.* 2023.

CITATION

Jourdain J, Barasc H, Faraut T, Calgaro A, Bonnet N, Marcuzzo C, Suin A, Barbat A, Hozé C, Besnard F, Taussat S, Grohs C, Kuchly C, Iampietro C, Donnadieu C, Pinton A, Boichard D, Capitan A (2023). Large-scale detection and characterization of interchromosomal rearrangements in normozoospermic bulls using massive genotype and phenotype data sets..

LINK

https://doi.org/10.1101/gr.277787.123

Citations

Step 1

Han JC & Han GY. A Procedure for Quantitative Determination of Tris(2-Carboxyethyl)phosphine, an Odorless Reducing Agent More Stable and Effective Than Dithiothreitol
https://doi.org/10.1006/abio.1994.1290

Step 1

Wu H, de Gannes MK, Luchetti G, Pilsner JR. Rapid method for the isolation of mammalian sperm DNA.
https://doi.org/10.2144/000114280

Step 9

Jourdain J, Barasc H, Faraut T, Calgaro A, Bonnet N, Marcuzzo C, Suin A, Barbat A, Hozé C, Besnard F, Taussat S, Grohs C, Kuchly C, Iampietro C, Donnadieu C, Pinton A, Boichard D, Capitan A. Large-scale detection and characterization of interchromosomal rearrangements in normozoospermic bulls using massive genotype and phenotype data sets.
https://doi.org/10.1101/gr.277787.123
