# Supplementary figures and images for: Validated DNA isolation method ensuring successful long-read sequencing of cattle semen genome
Source: PLoS One. 2024 Aug 7;19(8):e0308011. doi: 10.1371/journal.pone.0308011 (PMC11305549; doi:10.1371/journal.pone.0308011)

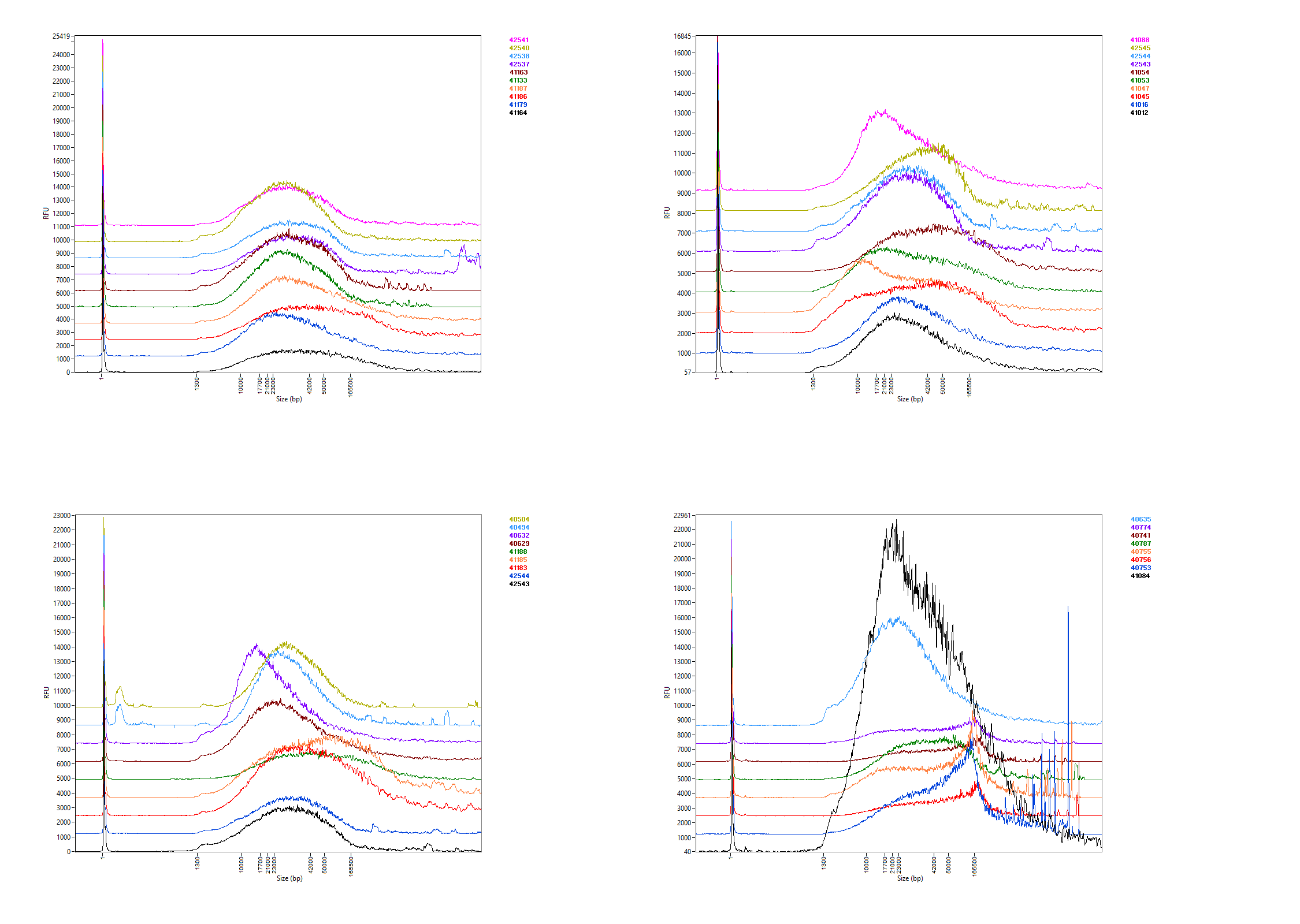

Supplement: S1 Fig — DNA fragment length distribution for 37 randomly selected samples (Fragment size distributions were assessed using the Femto pulse Genomic DNA 165 kb Kit (Agilent). Four different panels showed overlapping profiles of various DNA samples. (TIF) [file pone.0308011.s002.tif]

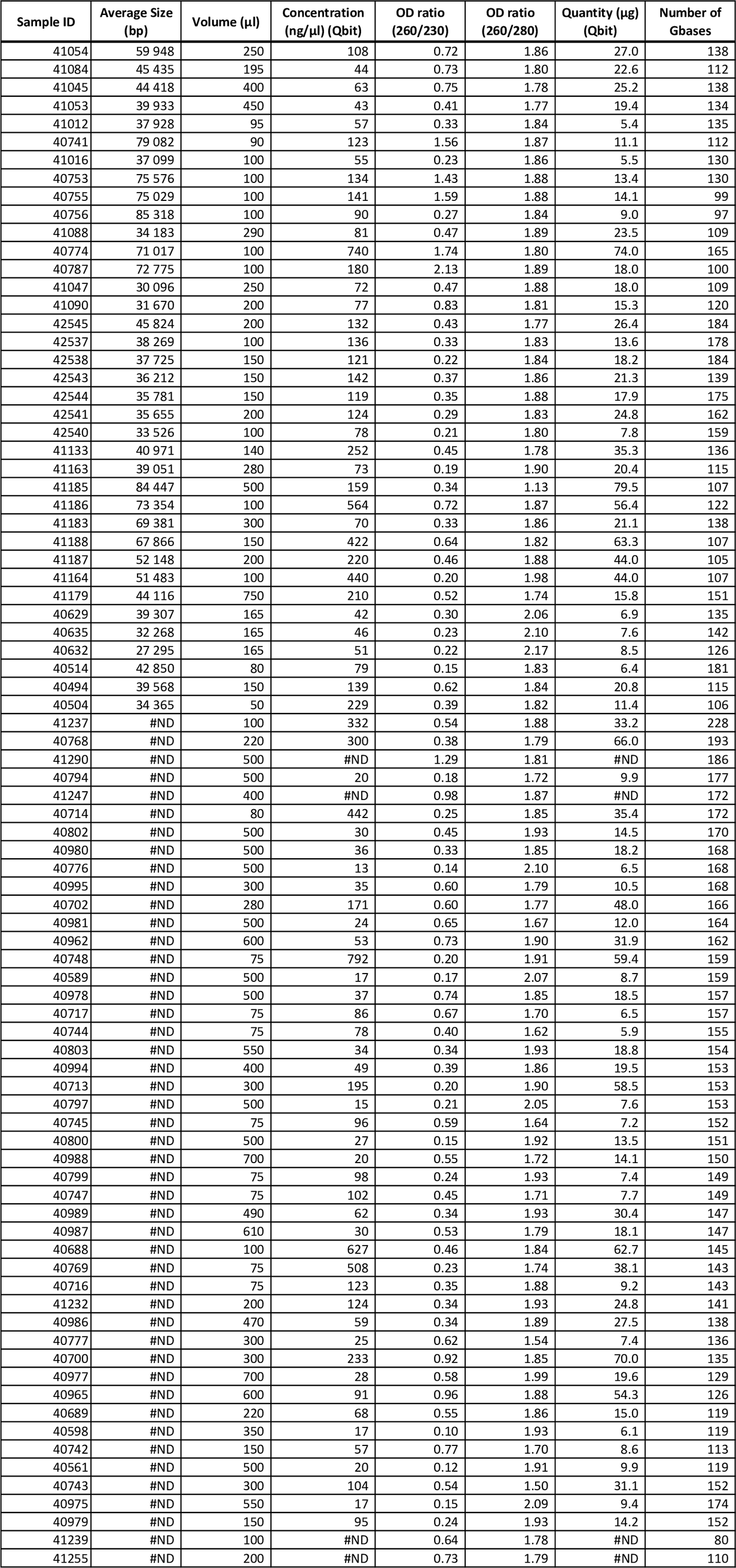

Supplement: S1 Table — Average size, volume, concentrations, OD ratios, quantities, and sequencing output (Gbases/SMRT cell) obtained for 84 samples. ND: No data. (TIF) [file pone.0308011.s003.tif]
